# Association of smoking cessation with airflow obstruction in workers with silicosis: A cohort study

**DOI:** 10.1371/journal.pone.0303743

**Published:** 2024-05-16

**Authors:** Shuyuan Yang, Chi Kuen Chan, Maggie Haitian Wang, Chi Chiu Leung, Lai Bun Tai, Lap Ah Tse

**Affiliations:** 1 Jockey Club School of Public Health and Primary Care, The Chinese University of Hong Kong, Hong Kong, Hong Kong SAR; 2 Department of Health, Tuberculosis and Chest Service, Hong Kong, Hong Kong SAR; 3 Stanley Ho Centre for Emerging Infectious Diseases, The Chinese University of Hong Kong, Hong Kong, Hong Kong SAR; China Medical University, CHINA

## Abstract

**Background:**

Studies in general population reported a positive association between tobacco smoking and airflow obstruction (AFO), a hallmark of chronic obstructive pulmonary disease (COPD). However, this attempt was less addressed in silica dust-exposed workers.

**Methods:**

This retrospective cohort study consisted of 4481 silicotic workers attending the Pneumoconiosis Clinic during 1981–2019. The lifelong work history and smoking habits of these workers were extracted from medical records. Spirometry was carried out at the diagnosis of silicosis (n = 4177) and reperformed after an average of 9.4 years of follow-up (n = 2648). AFO was defined as forced expiratory volume in one second (FEV_1_)/force vital capacity (FVC) less than lower limit of normal (LLN). The association of AFO with smoking status was determined using multivariate logistics regression, and the effect of smoking cessation on the development of AFO was evaluated Cox regression.

**Results:**

Smoking was significantly associated with AFO (current smokers: OR = 1.92, 95% CI 1.51–2.44; former smokers: OR = 2.09, 95% CI 1.65–2.66). The risk of AFO significantly increased in the first 3 years of quitting smoking (OR = 1.23, 95% CI 1.02–1.47) but decreased afterwards with increasing years of cessation. Smoking cessation reduced the risk of developing AFO no matter before or after the confirmation of silicosis (pre-silicosis cessation: HR = 0.58, 95% CI 0.46–0.74; post-silicosis cessation: HR = 0.62, 95% CI 0.48–0.79).

**Conclusions:**

Smoking cessation significantly reduced the risk of AFO in the workers with silicosis, although the health benefit was not observed until 3 years of abstinence. These findings highlight the importance of early and long-term smoking cessation among silicotic or silica dust-exposed workers.

## Introduction

Silicosis is one of the most important occupational diseases resulting from prolonged inhalation of respirable crystalline silica [1,2]. Although tremendous efforts have been made for decades to minimize this ancient and potentially fatal pneumoconiosis, failure to recognize and eliminate the silica-related exposure in some contemporary work practices (e.g., denim jean production and jewelry polishing) has leaded to a global re-emergence in recent years [1,3]. Chronic obstructive pulmonary disease (COPD) has been recognized as an irreversible disease characterized by persistent airflow obstruction, while it is preventable, it is highly prevalent in the silicotics [4,5] because a high proportion of silicotics were tobacco smoking with prolonged occupational exposure to respirable crystalline silica. Given the complex detrimental effects of particulate and irritant gaseous constituents of smoking on COPD, there is high biological plausibility that other inhaled toxins, e.g., respirable crystalline silica, also have an etiological role in the progression of this disease and thus increase the risk of incidence [6,7]. In fact, high prevalence of airflow obstruction (44% in ever smokers and 30% in never smokers) has been reported in a surveillance study of 1048 workers with silica exposure in Michigan [8], and higher likelihood of having airflow obstruction (OR = 3.06, 95% CI: 1.11–7.63) was also observed in the ELISABET study in French [9]. It is plausibly hypothesized that the harmful effect of smoking on the development of COPD in the workers with dust exposures may be worse than that in the general population [10,11].

Smoking cessation is the cornerstone of the treatment to COPD that not only improves the respiratory symptoms and quality of life, but also reduces the rate of pulmonary function decline and all-cause mortality [12]. Given the high prevalence of smoking among workers, the promotion of antitobacco and smoking cessation shall gain substantial health benefit in preventing the development of COPD. However, although a positive association of COPD with smoking and the benefit of smoking cessation were previously reported in the general population [13–16], few studies were focusing on the silicotic workers from the dusty trades, among whom the adverse effect of smoking may be potentiated by respirable crystalline silica or coexisting lung impairment [4]. Therefore, the aim of this study was to examine the association of spirometry-defined airflow obstruction (AFO), a hallmark of COPD, with self-reported smoking history (i.e., smoking status, pack-years, and time since quitting) and characterize the potential benefit of smoking cessation in a large occupational cohort of workers with silicosis.

## Materials and methods

### Study design and participants

This is a historical cohort study of silicotic workers recruited from the Pneumoconiosis Clinic, Hong Kong Department of Health during 1981–2019. Each worker took a physical examination including tuberculosis test, chest X-ray and spirometry at the diagnostic and follow-up assessments of silicosis. Detailed information on the history of silica-related occupational exposure and lifetime smoking habits was recorded by nurses. The diagnosis of silicosis was made by a medical panel based on workers’ relevant medical and occupational information along with chest radiographic changes (profusion subcategory 1/0 or higher) following the recommendation of the International Labor Organization (ILO) [17]. Workers with confirmed silicosis were invited for reassessment every 2 years for delineation of any additional disability and followed up till 31 December 2019. We extracted data of radiographic lung changes, lung function and smoking habits from medical records using a standard pro forma.‬‬‬‬‬‬‬‬‬‬‬‬‬‬‬‬‬‬‬‬‬‬‬‬‬‬‬‬‬‬‬‬‬‬‬‬‬‬‬‬‬‬‬‬‬‬‬‬‬‬‬‬‬‬‬‬‬‬‬‬ Eligible subjects of this study were 4481 workers diagnosed with silicosis during the study period. We excluded 304 workers who were aged over 80 or with missing or invalid data, and finally included 4177 in the current report. This study complies with the Declaration of Helsinki and was approved by the Survey and Behavior Research Ethics Committee of the Chinese University of Hong Kong (Reference No. SBRE-19-023).‬‬‬‬‬‬ The need of informed consent was waived by the ethics committee as this is a retrospective study of medical records and the data were analyzed anonymously.‬ Data of this study were accessed at the Pneumoconiosis Clinic from 1 January 2020 to 9 May 2021.‬‬‬‬‬‬‬‬‬‬‬‬‬‬‬‬‬‬‬‬‬‬‬‬‬‬‬‬‬‬‬‬‬‬

### Lung function

Lung function was measured using a dry wedge-type bellow spirometer (Vitalograph PFT II plus, Buckingham, UK), with the results corrected for body temperature, water vapor saturation and pressure. The European Respiratory Society guidelines were followed to ensure the validity and reproducibility of spirometry measurement. As recommended, three readings of forced expiratory volume in 1 second (FEV_1_) and forced vital capacity (FVC) from satisfactory maneuvers were obtained, and only the best one was recorded for statistical analyses. The predicted values and lower limits of normal (LLN) of FEV_1_, FVC and FEV_1_/FVC ratio adjusted for age, height, and sex were calculated using the reference equations developed by Hong Kong Thoracic Society [18]. AFO was defined as a FEV_1_/FVC ratio less than LLN.

### Tobacco smoking

**Fig 1:**
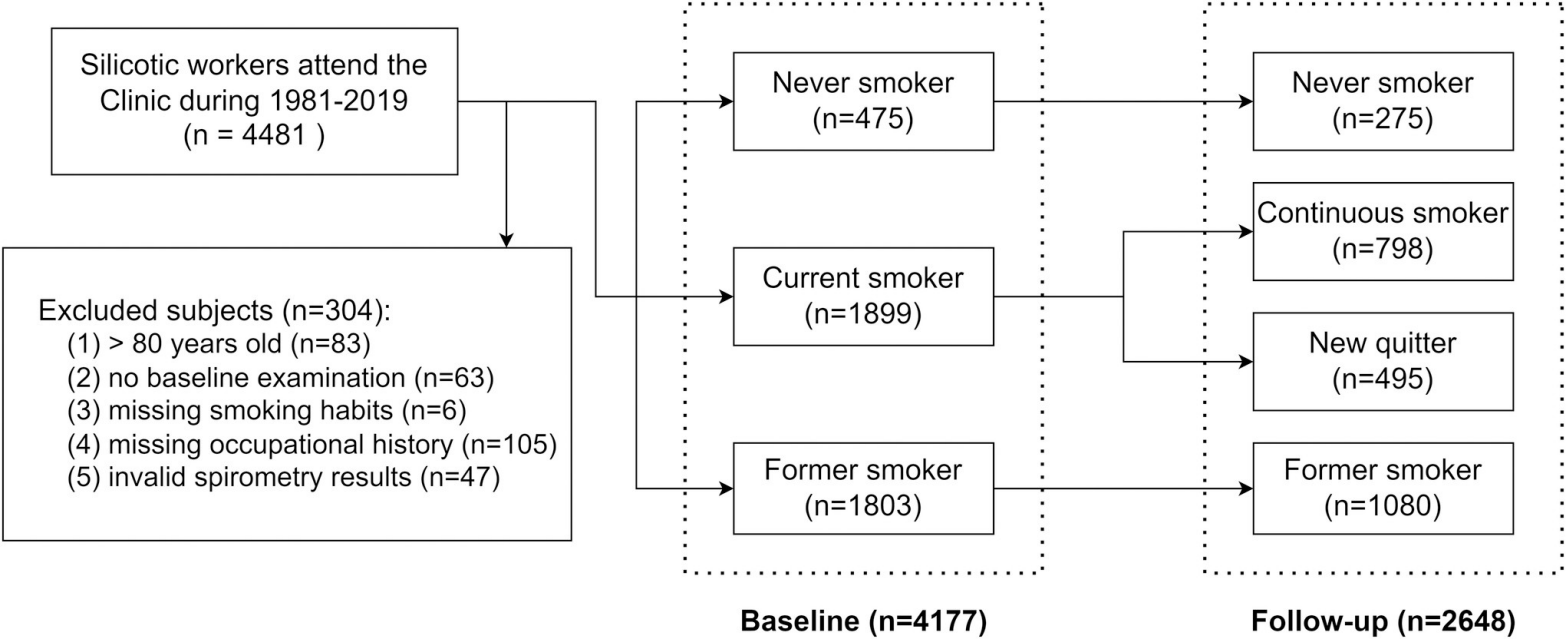
Flow diagram on the selection of study sample and the evolution of smoking status.

Tobacco smokers were defined as individuals who smoked no less than 100 cigarettes during their lifetime [19]. The smoking status at the diagnostic examination of silicosis was categorized as: (1) never smokers: never smoked or smoked less than 100 cigarettes during lifetime; (2) current smokers: smoked at the time of examination; and (3) former smokers: smoked before the examination but had quitted. The current smokers were further categorized as new quitters and continuous smokers according to the change of smoking habits during the follow-up period: new quitters gave up smoking during the follow-up, and continuous smokers kept smoking throughout the study period (Fig 1). The cumulative tobacco exposure was quantified as pack-years, which is the number of packs of cigarettes (20 per pack) smoked per day multiplied by years of smoking.

### Silica dust exposure

Silica dust exposure was assessed based on lifetime occupational history in combination with the job-exposure matrix. The comprehensive occupational history of each worker, including job duration, industry, title, task, type of dust exposed, was extracted from the medical records. The job-exposure matrix that comprises levels of exposure for corresponding industries and job tasks was constructed by referring the exposure level summarized by the US Occupational Safety and Health Administration [20]. After linking the individual occupation to the job-exposure matrix, the estimated silica dust exposure for each episode of job can be calculated by multiplying the exposure level of the certain job by job duration. The cumulative dust exposure was obtained by summing up the exposure of all episodes of jobs.

### Statistical analysis

Prevalence of AFO was calculated by dividing the number of AFO cases by the total number of workers. The baseline characteristics of workers with and without AFO were compared using Chi-square test. Comparison of lung function parameters by smoking status were carried out using Kruskal-Wallis test (nonparametric one-way ANOVA) with pairwise two-sided multiple comparison. The associations of smoking status, pack-years and time since cessation with AFO were examined using multivariate logistic regression, controlling for covariates including age, body mass index (BMI), history of tuberculosis, cumulative silica exposure and radiographic lung changes (cross-sectional analyses). The alpha level used in the multiple stepwise regression models of this study was pre-determined by setting an entry level of 0.05 and removal level of 0.1. Cox regression was used to elaborate the potential causal relationship between smoking and AFO based on the follow-up spirometry data, which is of a time-to-event nature (longitudinal analyses). AFO was redefined by fixed cut-off values (FEV_1_/FVC < 0.70) in a sensitivity analysis. All statistical analyses were carried out using SAS 9.4 (SAS Institute, Cary, North Carolina). A two-sided *p*-value less than 0.05 was considered statistically significant.

## Results

### Cohort characteristics

Among 4177 silica dust exposed workers, 3702 (89%) were ever smokers, including 1899 (46%) current smokers and 1803 (43%) former smokers (Table 1). Most of these workers were males (99%) with a median age of 56.1 (interquartile range: 48.7–63.5). A total of 1557 (37.3%) workers were found to have airflow obstruction at the diagnosis of silicosis. Compared with workers with non-obstructive spirometry, those with AFO had higher proportion of smoker (current smoker: 47% vs. 45%; former smoker: 46% vs. 42%), underweight (12% vs. 6%), ever tuberculosis (52% vs. 42%), progressive massive fibrosis (22% vs. 15%), and more smoking pack-years (mean pack-year: 28.8 vs. 23.0).

**Table 1:** Baseline characteristics of the workers by lung function category (n = 4177).

Characteristics	Total	Lung function category		χ^2^	*p*-value
		Non-AFO	AFO		
**Subjects No.**	4177 (100)	2620 (63)	1557 (37)		
**Age**					
**18–34**	35 (1)	23 (1)	12 (1)	10.4	0.02
**35–49**	1171 (28)	772 (30)	399 (26)		
**50–64**	2082 (50)	1258 (48)	824 (53)		
**≥ 65**	889 (21)	567 (22)	322 (21)		
**Sex**					
**Male**	4152 (9)	2602 (99)	1550 (100)	0.9	0.34
**Female**	25 (1)	18 (1)	7 (0)		
**BMI categories**					
**Underweight (<18.5)**	360 (9)	168 (6)	192 (12)	97.9	<0.001
**Normal (18.5–22.9)**	2068 (50)	1223 (47)	845 (54)		
**Overweight (23–24.9)**	909 (22)	618 (24)	291 (19)		
**Obese (≥25)**	840 (20)	611 (23)	229 (15)		
**Smoking status**					
**Never smoker**	475 (11)	360 (14)	115 (7.4)	39.6	<0.001
**Current smoker**	1899 (46)	1169 (45)	730 (47)		
**Former smoker**	1803 (43)	1091 (42)	712 (46)		
**Pack-years**					
**Never smoker**	475 (11)	360 (14)	115 (7)	71.0	<0.001
**Below 20**	1405 (34)	927 (36)	478 (31)		
**20 to 39**	1366 (33)	826 (32)	540 (35)		
**40 or above**	915 (22)	498 (19)	417 (27)		
**History of PTB**					
**Yes**	1896 (45)	1088 (42)	808 (52)	42.4	<0.001
**No**	2281 (55)	1532 (58)	749 (48)		
**Size of nodules**					
**Category p or s**	1747 (42)	1147 (44)	600 (39)	47.7	<0.001
**Category q or t**	2014 (49)	1274 (49)	740 (48)		
**Category r or u**	369 (9)	172 (7)	197 (13)		
**Profusion of nodules**					
**Category 1 (1/0, 1/1, 1/2)**	2294 (56)	1505 (58)	789 (51)	21.9	<0.001
**Category 2 (2/1, 2/2, 2/3)**	1487 (36)	900 (35)	587 (38)		
**Category 3 (3/2, 3/3, 3/+)**	345 (8)	187 (7)	158 (10)		
**Progressive massive fibrosis**					
**No (small opacites only)**	3411 (82)	2201 (85)	1210 (78)	26.9	<0.001
**Yes (with large opacity)**	731 (18)	397 (15)	334 (22)		
**Respiratory symptoms**					
**Cough**	2806 (67)	1689 (65)	1117 (72)	23.6	<0.001
**Dyspnea**	3264 (78)	1952 (75)	1312 (84)	55.1	<0.001
**Sputum**	2464 (59)	1436 (55)	1028 (66)	51.1	<0.001
**Chest pain**	1404 (34)	912 (35)	492 (32)	4.5	0.03
**Wheeze**	716 (17)	351 (13)	365 (24)	69.6	<0.001
**Hemoptysis**	392 (9)	224 (9)	168 (11)	5.8	0.02

Abbreviations: AFO, airflow obstruction; BMI, body mass index; PTB, pulmonary tuberculosis.

Values were presented as n (raw %), n (column %), or n (% yes).

### Lung function by smoking status

The mean FEV_1_, FVC and FEV_1_/FVC ratio in former smokers (FEV_1_% predicted = 79%, FVC % predicted = 90%, FEV_1_/FVC = 0.69) were lower than those in current smokers (FEV_1_% predicted = 84%, FVC % predicted = 93%, FEV_1_/FVC = 0.71) at baseline. Never smokers had better FEV_1_ and FEV_1_/FVC but similar FVC (FEV_1_% predicted = 86%, FVC % predicted = 90%, FEV_1_/FVC = 0.75) as compared with ever smokers (S1 Table). Results of the follow-up spirometry revealed that the lung function of new quitters who gave up smoking after baseline (FEV_1_% predicted = 64%, FVC % predicted = 80%, FEV_1_/FVC = 0.61) were worse than that of the former smokers (FEV_1_% predicted = 68%, FVC % predicted = 82%, FEV_1_/FVC = 0.63) and even the continuous smokers (FEV_1_% predicted = 69%, FVC % predicted = 83%, FEV_1_/FVC = 0.64) (S2 Table). Differences in lung function indices between smoking groups were statistically significant.

### Association of AFO with smoking status and pack-years

Smokers, including both former smokers and current smokers, had an increased risk of AFO compared with non-smokers (Table 2). The risk in former smokers (crude OR = 2.09, 95% CI 1.65–2.66) was higher than that in the current smokers (crude OR = 1.92, 95% CI 1.51–2.44). After adjusting for the covariates, the difference in the risk of AFO between the former smokers (adjusted OR = 1.82, 95% CI 1.42–2.33) and current smokers (adjusted OR = 1.64; 95% CI 1.28–2.10) became attenuated, but the risk of former smokers remained higher than that in the current smokers. There was no significant interaction between smoking status and cumulative silica exposure on the risk of AFO (*p* for interaction = 0.17).

**Table 2:** Association of airflow obstruction (AFO) with smoking status and pack-years in workers with silicosis (n = 4177).

Smoking status & pack-years	No.	No. (%) with AFO	Crude OR (95% CI) Model 0	Adjusted OR (95% CI)		
				Model 1	Model 2	Model 3
**Never smoker**	475	115 (24)	1.00 (Ref.)	1.00 (Ref.)	1.00 (Ref.)	1.00 (Ref.)
**Current smoker**	1899	730 (38)	1.92 (1.51, 2.44)	1.72 (1.34, 2.19)	1.71 (1.34, 2.18)	1.64 (1.28, 2.10)
**Below 20**	658	218 (33)	1.48 (1.12, 1.96)	1.37 (1.04, 1.83)	1.36 (1.03, 1.81)	1.32 (0.99, 1.76)
**20 to 39**	785	309 (39)	1.98 (1.52, 2.58)	1.73 (1.32, 2.27)	1.72 (1.31, 2.26)	1.67 (1.27, 2.20)
**40 or more**	453	201 (44)	2.49 (1.86, 3.35)	2.19 (1.62, 2.96)	2.18 (1.61, 2.94)	2.06 (1.52, 2.80)
**Former smoker**	1803	712 (40)	2.09 (1.65, 2.66)	1.91 (1.49, 2.44)	1.89 (1.48, 2.41)	1.82 (1.42, 2.33)
**Below 20**	747	260 (35)	1.63 (1.25, 2.13)	1.54 (1.17, 2.02)	1.52 (1.15, 2.00)	1.48 (1.12, 1.95)
**20 to 39**	581	231 (40)	2.23 (1.68, 2.95)	2.02 (1.52, 2.70)	2.01 (1.51, 2.68)	1.95 (1.46, 2.61)
**40 or more**	462	216 (47)	3.10 (2.30, 4.19)	2.67 (1.96, 3.63)	2.63 (1.94, 3.58)	2.50 (1.83, 3.42)

Abbreviations: AFO, airflow obstruction; OR, odds ratio; CI, confidence interval; LLN, lower limit of normal; BMI, body mass index; FEV_1_, forced expiratory volume in 1 second; FVC, forced vital capacity.

Airflow obstruction was defined as FEV_1_/FVC ratio < LLN.

Model 0: Controlled for age at baseline only.

Model 1: Model 0 adjusted for history of pulmonary tuberculosis and BMI category.

Model 2: Model 1 adjustments plus cumulative silica exposure.

Model 3: Model 2 adjustments plus the size and profusion of small opacities in lung and progressive massive fibrosis.

Smoking pack-years were positively associated with the AFO prevalence at the baseline. Compared with never smokers, the crude OR in current smokers was 1.48 (95% CI 1.12–1.96) in those who smoked less than 20 pack-years but increased to 1.98 (95% CI 1.52–2.58) in those smoked 20–39 pack-years and 2.49 (95% CI 1.86–3.35) in those smoked 40 pack-years or more. These associations became more pronounced in former smokers. These associations remained significant after controlling for age, body mass index (BMI), history of tuberculosis, cumulative silica exposure and radiographic signs of silicotic nodules.

### Association of AFO with time since quitting

In smokers who gave up smoking during the follow-up (i.e., new quitters), the time since quitting was negatively associated with AFO, but the relationship was not linear (Table 3). Compared with the current smokers, former smokers quitting smoking within 3 years had significantly increased risk of AFO (adjusted OR = 1.23, 95% CI 1.02–1.47). Among the former smokers, the magnitude of OR remained above 1.00 until 9 years after quitting smoking and showed an insignificant decrease to 0.82 (95% CI 0.62–1.10) for 15+ years of cessation. However, a significantly reduced risk of AFO was only observed among never smokers (adjusted OR = 0.61, 95% CI: 0.47–0.78).

**Table 3:** Association of airflow obstruction (AFO) with time since quitting in workers with silicosis (n = 4177).

Time since quitting	No.	No. (%) with AFO	Crude OR (95% CI) Model 0	Adjusted OR (95% CI)		
				Model 1	Model 2	Model 3
**0 (current smoker)**	1899	730 (38)	1.00 (Ref.)	1.00 (Ref.)	1.00 (Ref.)	1.00 (Ref.)
**Below 3 years**	879	370 (42)	1.29 (1.09, 1.54)	1.25 (1.04, 1.49)	1.24 (1.04, 1.48)	1.23 (1.02, 1.47)
**3 to 5.9 years**	280	115 (41)	1.14 (0.87, 1.49)	1.17 (0.89, 1.54)	1.17 (0.88, 1.54)	1.16 (0.88, 1.54)
**6 to 8.9 years**	140	53 (38)	1.07 (0.73, 1.57)	1.19 (0.81, 1.76)	1.19 (0.80, 1.75)	1.21 (0.82, 1.80)
**9 to 11.9 years**	142	53 (37)	0.92 (0.64, 1.35)	0.95 (0.65, 1.40)	0.95 (0.65, 1.40)	0.96 (0.65, 1.42)
**12 to 14.9 years**	63	22 (35)	0.75 (0.43, 1.30)	0.85 (0.48, 1.49)	0.82 (0.47, 1.44)	0.85 (0.48, 1.50)
**15 years or more**	299	99 (33)	0.71 (0.54, 0.93)	0.81 (0.61, 1.07)	0.80 (0.60, 1.06)	0.82 (0.62, 1.10)
**NA (never smoker)**	475	115 (24)	0.52 (0.41, 0.66)	0.58 (0.45, 0.74)	0.58 (0.46, 0.74)	0.61 (0.47, 0.78)

Abbreviations: AFO, airflow obstruction; OR, odds ratio; CI, confidence interval; LLN, lower limit of normal; BMI, body mass index; FEV_1_, forced expiratory volume in 1 second; FVC, forced vital capacity.

Airflow obstruction was defined as FEV_1_/FVC ratio < LLN.

Model 0: Controlled for age at baseline only.

Model 1: Model 0 adjusted for history of pulmonary tuberculosis and BMI category.

Model 2: Model 1 adjustments plus cumulative silica exposure.

Model 3: Model 2 adjustments plus the size and profusion of small lung opacities and progressive massive fibrosis.

### Effect of smoking cessation on the development of AFO

**Fig 2:**
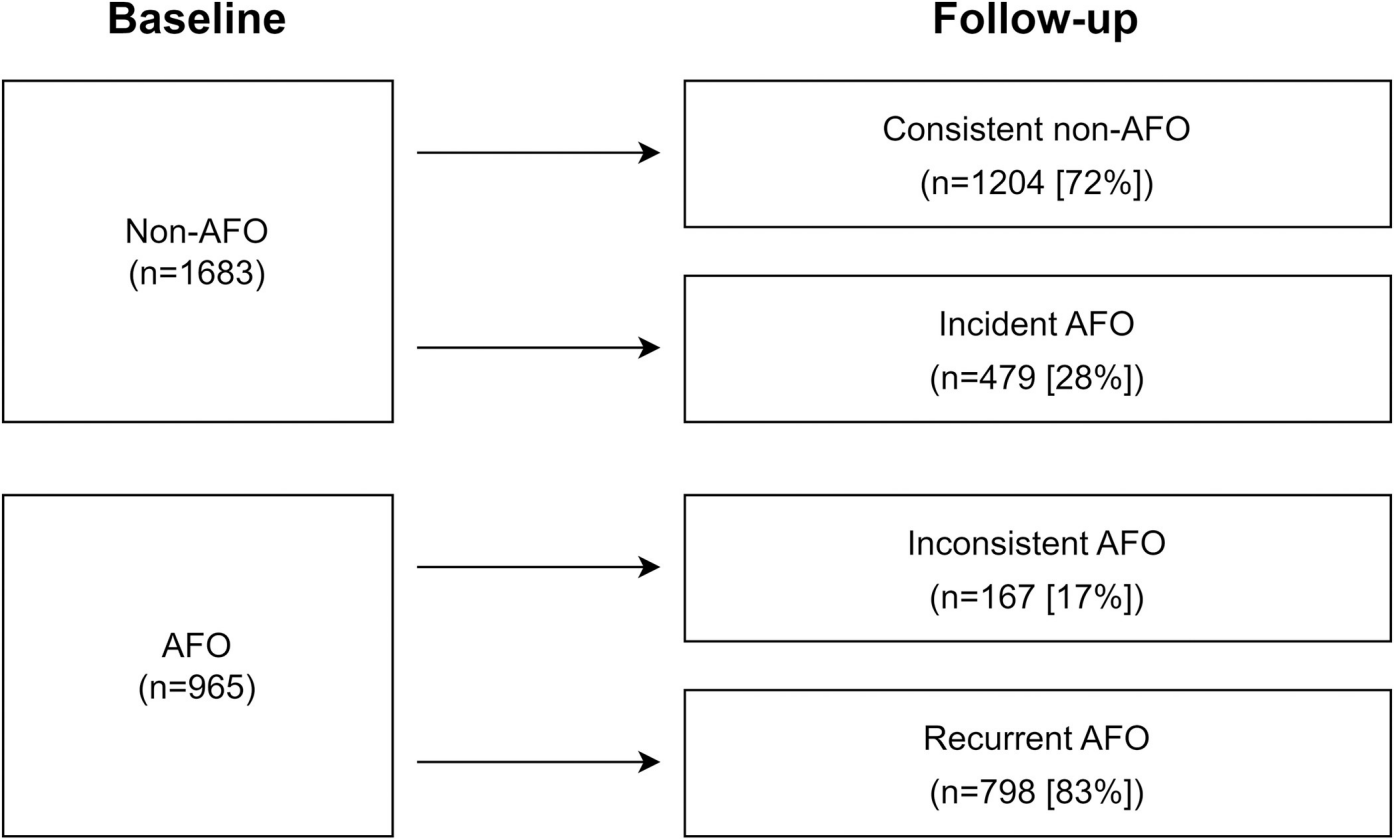
Transition of lung function categories Consistent non-AFO, nonobstructive spirometry at baseline and follow-up; Incident AFO, nonobstructive spirometry at baseline but AFO at follow-up; Inconsistent AFO, AFO at baseline but nonobstructive spirometry at follow-up; Recurrent AFO, AFO at baseline and follow-up. Abbreviations: AFO, airflow obstruction.

In this cohort of workers with silicosis, a total of 2648 subjects had a reassessed spirometry after an average of 9.4 years. These workers tended to be younger, with a higher prevalence of smoking but less pack-years than those without follow-up spirometry (S3 Table). All workers had the same hazards at the diagnosis of silicosis. There were 1293 current smokers included in the longitudinal data, among them 495 (38.3%) workers quit smoking afterwards, and 798 (61.7%) workers kept their smoking habits throughout the study period. The prevalence of AFO among these workers was 36% (n = 965), which was not significantly different from that in workers without follow-up spirometry (*p* = 0.14). Among the remaining 1683 subjects with non-obstructive spirometry at baseline, 479 (28%) were identified as the new AFO in the follow-up assessment (Fig 2). Compared with the continuous smokers, both new quitters and former smokers had significantly reduced hazard of developing new AFO events (new quitters: HR = 0.62, 95% CI 0.48–0.79; former smokers: HR = 0.58, 95% CI 0.46–0.74), and the never smokers had the lowest risk of AFO (HR = 0.57, 95% CI 0.41–0.81) (Table 4).

**Table 4:** Hazard ratios and 95% confidence intervals for the development of airflow obstruction (AFO) in workers with non-obstructive spirometry at baseline (n = 1683).

Smoking status during follow-up	No.	No. (%) with AFO	Crude HR (95% CI) Model 0	Adjusted HR (95% CI)		
				Model 1	Model 2	Model 3
**Continuous smoker**	493	153 (31)	1.00 (Ref.)	1.00 (Ref.)	1.00 (Ref.)	1.00 (Ref.)
**New quitter**	317	128 (40)	0.62 (0.48, 0.79)	0.63 (0.50, 0.81)	0.63 (0.49, 0.81)	0.62 (0.48, 0.79)
**Former smoker**	665	153 (23)	0.61 (0.49, 0.77)	0.61 (0.48, 0.76)	0.60 (0.48, 0.76)	0.58 (0.46, 0.74)
**Never smoker**	208	45 (22)	0.56 (0.40, 0.79)	0.58 (0.42, 0.82)	0.59 (0.42, 0.82)	0.57 (0.41, 0.81)

Abbreviations: AFO, airflow obstruction; HR, hazard ratio; CI, confidence interval; LLN, lower limit of normal; BMI, body mass index; FEV_1_, forced expiratory volume in 1 second; FVC, forced vital capacity.

Airflow obstruction was defined as FEV_1_/FVC ratio < LLN.

Model 0: Controlled for age at baseline only.

Model 1: Model 0 adjusted for history of pulmonary tuberculosis and BMI category.

Model 2: Model 1 adjustments plus cumulative silica exposure.

Model 3: Model 2 adjustments plus the size and profusion of small lung opacities and progressive massive fibrosis.

### Sensitivity analysis

The associations of AFO with smoking status, pack-years, and time since quitting, and the effect of smoking cessation on the incident AFO were robust when AFO was defined by a fixed cut-off value (FEV_1_/FVC < 0.7).

## Discussion

In this large historical cohort study of 4117 silicotic workers in Hong Kong, tobacco smoking was positively associated with the presence of AFO, a hallmark of COPD. The higher risk of AFO was observed in smokers with more pack-years, and a long-term benefit in reducing the risk of AFO was indicated among workers who quit smoking. Of note, the significantly elevated risk of AFO in quitters retained in the first 3 years after smoking cessation but the benefit of abstinence revealed after 9 years. However, the longitudinal analyses revealed that smoking cessation significantly reduced the risk of AFO. These associations remained unchanged after a full adjustment of covariates including age, body mass index, pulmonary tuberculosis, silica dust exposure and lung opacities.

Tobacco smoking is a well-established risk factor of COPD. The positive association between smoking and COPD has been identified in several population-based epidemiological studies, e.g., the Canadian Obstructive Lung Disease Study [21] and the China Pulmonary Health Study [22]. However, a recent cross-sectional study comprised of 675 patients with pneumoconiosis provided some indication for a potential interaction between smoking and silica dust exposure on the incident COPD [4]. The potential excess risk of COPD observed among smoking silicotics may be explained by the progression of pulmonary fibrosis induced by prolonged exposure to silica dust, which may lead to large airway narrowing and distortion and small airway dysfunction and eventually cause the formation of COPD [23]. In our study on a clinically defined cohort of silicotic workers, we reported significantly lower FEV_1_, FEV_1_/FVC ratio and a nearly one-fold increase in the risk of AFO among smokers compared with the never smokers. These findings demonstrated the significant relationship between AFO and smoking among workers with silicosis, with an association being consistent with that was reported in the previous epidemiological studies in general populations [21,22]. We also investigated the potential interactions between smoking and cumulative dust exposure in relation to AFO but no evidence of interaction was found, which is consistent with the findings from Xingtai nested case-control study [24] and Guangzhou Biobank Cohort Study [25]. However, conflicting findings were reported in some other studies, which suggest a significant interaction between smoking and dust exposure in the association with AFO [26,27]. Since the mechanisms on how dust exposure and smoking work on the development of AFO is still unclear, further research on this specific mechanism is warranted.

Smoking cessation is considered as the most effective intervention that slows down pulmonary function decline and prevents the development of COPD [28]. Quitting smoking for 12 months was found to have 42% reduction of conductive airways malfunction, which was typically observed prior to AFO [29,30]. A reduced risk of AFO in the smoking quitters with increased time since quitting was also observed in this study. However, the magnitude of OR remained no significant reduction in the first 9 years, suggesting the presence of “quit ill effect” that the benefit from smoking cessation among silicotic workers was not initiated immediately after quitting smoking but indicated a time window of at least 3 years [31]. A potential explanation of this phenomenon is the “healthy smoker” theory that the individuals due to poor health tend to avoid or quit smoking, while smokers with good health condition (e.g., having fewer respiratory symptoms) are not motivated to abstain [32,33]. In this cohort of workers with silicosis, we observed poorer lung function (i.e., lower FEV_1_ and FEV_1_/FVC ratio) in the former smokers than in the current smokers, suggesting some smokers were more prone to cessation because they suffered from the respiratory symptoms or other ill-being caused by the impaired pulmonary functionality, e.g., cough or wheezing, rather than motivated by the pursuit of health. These quitters may thus have increased likelihood of AFO that mask the benefit of quitting smoking in the subsequent several years after abstinence. However, after cessation for long time, the benefit of quitting smoking on the reduction of AFO was evidently manifested.

The significantly reduced risk of AFO among the quitters with non-obstructive spirometry further supported the probability of ‘quit ill effect’ of smokers in this occupational cohort. Results from the cross-sectional analyses indicated an increased risk of AFO within 3 years of smoking cessation and a steadily decreasing trend of risk afterwards, although the risk of quitters could not return to the background level as that of the never smokers. Given the benefit of smoking cessation is well identified and widely acknowledged, it is incredible to attribute this extra detrimental effect to smoking cessation. The phenomenon of ‘quit ill effect’ may explain the potential reverse causality between smoking cessation and AFO, as the silicotic workers with a habit of smoking probably would have to quit smoking due to the presence of intolerable respiratory symptoms or other ill-being caused by the impaired spirometry during the follow-up. In the longitudinal analyses, as the significant benefit of smoking cessation revealed when excluding the workers with AFO at baseline, the beneficial effect of smoking cessation was confirmed. These findings supported the benefit of smoking cessation and highlighted the cautions in making a proper interpretation to the results obtained solely from cross-sectional analyses, in which the causality cannot be well determined.

The strengths of this study include a large sample size with a territory-wide coverage of study population, high-quality spirometry data, a long period of follow-up over 39 years and the integrity of data on radiographic lung changes, work history and smoking habits. However, some potential limitations should be considered. Firstly, although the nurses at Pneumoconiosis Clinic regularly contacted the silicotic workers, not all these workers returned to the Clinic and took the routine reassessment. Compared with those without a follow-up assessment, the reassessed workers were younger (aged over 65: 12% vs. 38%), with lower proportion of underweight (7% vs. 12%) but more current smokers (50% vs. 37%), suggesting that the association of smoking with AFO might be underestimated in the longitudinal analyses of this study. Secondly, concerns were raised on potential confounding effects of outdoor PM2.5 air pollution and household air pollution (e.g., second-hand smoking, cooking fuels), as this information was not collected by this retrospective cohort study. We checked the home addresses of the participating silicotic workers from the clinical records and found most of them lived in the public household where town gas is routinely supplied for cooking. As the air quality of Hong Kong is mainly affected by regional aerosol emissions, variations in PM2.5 concentrations were similar in different areas of this city [34]. Since 2009, public areas of Hong Kong have implemented the statutory smoking ban regulations which largely reduced workers’ exposures to second-hand smoking. Hence, potential confounding of both outdoor and indoor air pollution on the effect of AFO should not be a major issue of our study. Thirdly, although the current data have already achieved the primary aim of this study, which was to investigate the association of smoking and cessation with the development of AFO among workers with silicosis, lack of data from the workers without silicosis made it unable to test whether such association among those without silicosis. A future study comparing the relationship between smoking and AFO among workers with and without silicosis is warranted when the counterpart data from non-silicosis workers are available.

In summary, the present study demonstrated a “quit ill effect” in the workers with silicosis that the benefit of smoking cessation on the reduction of AFO may not initiate immediately after the time of quitting but would demonstrate after 3 years of abstinence. These findings deliver positive messages to the dust-exposed or silicotic workers that smoking cessation shall be long-term to be beneficial. In view of the high prevalence of smoking among the dust-exposed workers, there is a pressing need for advocating early and long-term smoking cessation to prevent the development of COPD.

## Supporting information

S1 Table Baseline lung function of the silicotic workers (n = 4177) by smoking status at baseline. (DOCX)

S2 Table Follow-up lung function of the silicotic workers (n = 2648) by smoking status at follow-up. (DOCX)

S3 Table Characteristics of silicotic workers with and without follow-up spirometry. (DOCX)
