# Supplementary material for: Association of smoking cessation with airflow obstruction in workers with silicosis: A cohort study
Source: PLoS One. 2024 May 16;19(5):e0303743. doi: 10.1371/journal.pone.0303743 (PMC11098359; doi:10.1371/journal.pone.0303743)
Supplement: S1 Table — (DOCX) [file pone.0303743.s001.docx]

**S1 Table. Baseline lung function of the silicotic workers (n=4177) by smoking status at baseline**

|  | **Never smoker** | **Former smoker** | **Current smoker** | ***p*-value** | **Pairwise significance** | | | |
| --- | --- | --- | --- | --- | --- | --- | --- | --- |
|  |  |  |  |  | **NS vs FS** | **NS vs CS** | **FS vs CS** |  |
| **FEV_1_ (L/second)** | 2.22 | 2.05 | 2.25 | <0.001 | <0.001 | 0.87 | <0.001 |  |
| **FEV_1_, % predicted** | 86 | 79 | 84 | <0.001 | <0.001 | 0.13 | <0.001 |  |
| **FVC (L)** | 2.94 | 2.94 | 3.14 | <0.001 | 0.86 | <0.001 | <0.001 |  |
| **FVC, % predicted** | 90 | 90 | 93 | <0.001 | 0.80 | <0.001 | <0.001 |  |
| **FEV_1_/FVC ratio** | 0.75 | 0.69 | 0.71 | <0.001 | <0.001 | <0.001 | <0.001 |  |

Abbreviations: NS, never smoker; FS, former smoker; CS, current smoker; FEV_1_, forced expiratory volume in 1 second; FVC, forced vital capacity.

Means were compared using Kruskal-Wallis test.
