# Supplementary material for: Association of smoking cessation with airflow obstruction in workers with silicosis: A cohort study
Source: PLoS One. 2024 May 16;19(5):e0303743. doi: 10.1371/journal.pone.0303743 (PMC11098359; doi:10.1371/journal.pone.0303743)
Supplement: S2 Table — (DOCX) [file pone.0303743.s002.docx]

**S2 Table. Follow-up lung function of the silicotic workers (n=2648) by smoking status at follow-up**

|  | **Never smoker** | **Former smoker** | **New quitter** | **Continuous smoker** | ***p*-value** |
| --- | --- | --- | --- | --- | --- |
| **FEV_1_** | 1.75 | 1.65 | 1.55 | 1.71 | <0.001 |
| **FEV_1_ % predicted** | 73 | 68 | 64 | 69 | <0.001 |
| **FVC** | 2.52 | 2.58 | 2.53 | 2.63 | 0.03 |
| **FVC % predicted** | 81 | 82 | 80 | 83 | 0.05 |
| **FEV_1_/FVC ratio** | 0.69 | 0.63 | 0.61 | 0.64 | <0.001 |

Abbreviations: FEV_1_, forced expiratory volume in 1 second; FVC, forced vital capacity.
