# Supplementary material for: Association of smoking cessation with airflow obstruction in workers with silicosis: A cohort study
Source: PLoS One. 2024 May 16;19(5):e0303743. doi: 10.1371/journal.pone.0303743 (PMC11098359; doi:10.1371/journal.pone.0303743)
Supplement: S3 Table — (DOCX) [file pone.0303743.s003.docx]

**S3 Table. Characteristics of silicotic workers with and without follow-up spirometry**

| **Characteristics** | **Total** | **Follow-up spirometry** | |  |
| --- | --- | --- | --- | --- |
|  |  | **Yes** | **No** | ***p*-value** |
| **Subjects No.** | 4177 (100) | 2648 (63) | 1529 (37) |  |
| **Age** |  |  |  |  |
| **18-34** | 35 (1) | 28 (1) | 7 (0) | <0.001 |
| **35-49** | 1171 (28) | 934 (35) | 237 (16) |  |
| **50-64** | 2082 (50) | 1371 (52) | 711 (47) |  |
| **≥ 65** | 889 (21) | 315 (12) | 574 (38) |  |
| **Sex** |  |  |  |  |
| **Male** | 4152 (99) | 2637 (100) | 1515 (99) | 0.04 |
| **Female** | 25 (1) | 11 (0) | 14 (1) |  |
| **BMI categories** |  |  |  |  |
| **Underweight (<18.5)** | 360 (9) | 182 (7) | 178 (12) | <0.001 |
| **Normal (18.5-22.9)** | 2068 (49) | 1359 (51) | 709 (46) |  |
| **Overweight (23-24.9)** | 909 (22) | 582 (22) | 327 (21) |  |
| **Obese (≥25)** | 840 (20) | 525 (20) | 315 (21) |  |
| **Smoking status** |  |  |  |  |
| **Never smoker** | 475 (11) | 275 (10) | 200 (13) | <0.001 |
| **Current smoker** | 1899 (46) | 1328 (50) | 571 (37) |  |
| **Former smoker** | 1803 (43) | 1045 (40) | 758 (50) |  |
| **Pack-years** |  |  |  |  |
| **Never smoker** | 475 (11) | 275 (10) | 200 (13) | <0.001 |
| **Below 20** | 1405 (34) | 949 (36) | 456 (30) |  |
| **20 to 39** | 1366 (33) | 919 (35) | 447 (29) |  |
| **40 or more** | 915 (22) | 498 (19) | 417 (27) |  |
| **History of PTB** |  |  |  |  |
| **Yes** | 1896 (45) | 1196 (45) | 700 (46) | 0.70 |
| **No** | 2281 (55) | 1452 (55) | 829 (54) |  |
| **Size of nodules** |  |  |  |  |
| **Category p or s** | 1747 (42) | 996 (38) | 751 (50) | <0.001 |
| **Category q or t** | 2014 (49) | 1389 (53) | 625 (41) |  |
| **Category r or u** | 369 (9) | 235 (9) | 134 (9) |  |
| **Profusion of nodules** |  |  |  |  |
| **Category 1 (1/0, 1/1, 1/2)** | 2294 (56) | 1261 (48) | 1033 (68) | <0.001 |
| **Category 2 (2/1, 2/2, 2/3)** | 1487 (36) | 1096 (42) | 391 (26) |  |
| **Category 3 (3/2, 3/3, 3/+)** | 345 (8) | 255 (10) | 90 (6) |  |
| **Progressive massive fibrosis** |  |  |  |  |
| **No (small opacites only)** | 3411 (82) | 2164 (82) | 1247 (82) | 0.85 |
| **Yes (with large opacity)** | 731 (18) | 461 (18) | 270 (18) |  |
| **Respiratory symptoms** |  |  |  |  |
| **Cough** | 2806 (67) | 1757 (66) | 1049 (69) | 0.14 |
| **Dyspnea** | 3264 (78) | 2066 (78) | 1198 (78) | 0.82 |
| **Sputum** | 2464 (59) | 1519 (57) | 945 (62) | 0.005 |
| **Chest pain** | 1404 (34) | 903 (34) | 501 (33) | 0.37 |
| **Wheeze** | 716 (17) | 377 (14) | 339 (22) | <0.001 |
| **Hemoptysis** | 392 (9) | 211 (8) | 181 (12) | <0.001 |

Abbreviations: BMI, body mass index; PTB, pulmonary tuberculosis.

Values were presented as n (raw %), n (column %), or n (% yes).
